# Magnetoreception Regulates Male Courtship Activity in *Drosophila*

**DOI:** 10.1371/journal.pone.0155942

**Published:** 2016-05-19

**Authors:** Chia-Lin Wu, Tsai-Feng Fu, Meng-Hsuan Chiang, Yu-Wei Chang, Jim-Long Her, Tony Wu

**Affiliations:** 1 Department of Biochemistry, College of Medicine, Chang Gung University, Taoyuan, 33302, Taiwan; 2 Graduate Institute of Biomedical Sciences, College of Medicine, Chang Gung University, Taoyuan, 33302, Taiwan; 3 Molecular Medicine Research Center, Chang Gung University, Taoyuan, 33302, Taiwan; 4 Department of Neurology, Linkou Chang Gung Memorial Hospital, Taoyuan, 33305, Taiwan; 5 Department of Applied Chemistry, National Chi-Nan University, Nantou, 54561, Taiwan; 6 Division of Natural Science, Center for General Education, Chang Gung University, Taoyuan, 33302, Taiwan; Academia Sinica, TAIWAN

## Abstract

The possible neurological and biophysical effects of magnetic fields on animals is an area of active study. Here, we report that courtship activity of male *Drosophila* increases in a magnetic field and that this effect is regulated by the blue light-dependent photoreceptor cryptochrome (CRY). Naïve male flies exhibited significantly increased courtship activities when they were exposed to a ≥ 20-Gauss static magnetic field, compared with their behavior in the natural environment (0 Gauss). CRY-deficient flies, *cry*^*b*^ and *cry*^*m*^, did not show an increased courtship index in a magnetic field. RNAi-mediated knockdown of *cry* in *cry-GAL4*-positive neurons disrupted the increased male courtship activity in a magnetic field. Genetically expressing *cry* under the control of *cry-GAL4* in the CRY-deficient flies restored the increase in male courtship index that occurred in a magnetic field. Interestingly, artificially activating *cry-GAL4-*expressing neurons, which include large ventral lateral neurons and small ventral lateral neurons, via expression of thermosensitive cation channel *dTrpA1*, also increased the male courtship index. This enhancement was abolished by the addition of the *cry-GAL80* transgene. Our results highlight the phenomenon of increased male courtship activity caused by a magnetic field through CRY-dependent magnetic sensation in CRY expression neurons in *Drosophila*.

## Introduction

All organisms on Earth are exposed to the planet’s natural magnetic field, which is approximately 0.3–0.5 Gauss (G). Some animals use this geomagnetic field for navigation and orientation [1–3]. Three modes of magnetoreception have been proposed in the past [4]. First, it has been suggested that electromagnetic induction by the geomagnetic field occurs in marine animals; however, there is little evidence to support this model. Second, the magnetite-based hypothesis proposes that magnetoreception occurs via tiny crystals of permanent ferromagnetic materials [5]. Third, the chemical reaction model proposes that magnetic information is transmitted to the nervous system via light-dependent products and relies on magnetically sensitive radical-pair reactions in specialized photoreceptors [6].

Magnetoreception is a wavelength-dependent process that occurs via cryptochromes (CRYs) in birds and fruit flies (*Drosophila melanogaster*) [7,8]. CRYs are flavoproteins that are sensitive to light in the ultraviolet and blue ranges and contain photoactivatable flavin adenine dinucleotide (FAD) chromophores that form radical pairs following blue light activation [9]. CRYs are expressed in the retinas of migratory birds and may function in the performance of nocturnal magnetic-orientation tasks [10]. In fruit flies, there is only one CRY, and it is expressed in the circadian clock neurons of the brain [11,12]. CRY-mediated light-dependent magnetosensitivity has been reported to influence the *Drosophila* circadian clock [13]. Under blue-light conditions, flies demonstrated slowing of the circadian clock when a static magnetic field was applied [13]. In addition, under a magnetic field, flies that overexpressed *cry* in clock neurons enhanced the length of their period, whereas *cry* mutants showed no response. Thus, the *Drosophila* circadian clock is sensitive to light-mediated CRY activation and to magnetic fields, which is consistent with the radical-pair mechanism [13].

To determine whether transient exposure to a magnetic field also affects fly courtship behaviors, we devised a Helmholtz coil-type apparatus that produced a stable magnetic field between two coils. Using the apparatus, *Drosophila melanogaster* courtship behaviors were analyzed at different magnetic field strengths. Wild-type flies, including white-eyed Canton-S, red-eyed Oregon-R, and red-eyed Canton-S flies, all showed increased courtship activities in response to the enhanced magnetic field (≥ 20 G). The increase in the courtship index (the percentage of time a male spends courting a female) decreased when < 500 nm wavelength light was blocked, suggesting that this behavioral phenotype is blue light-dependent [8].

In the fruit fly, light-dependent magnetosensitivity requires the blue-light photoreceptor CRY [8]. Two *cry* mutant flies (*cry*^*b*^ and *cry*^*m*^) did not show increased courtship indices in the magnetic field environments. Targeted dsRNA-mediated silencing of *cry* in *cry-GAL4*-positive neurons also eliminated the increase in courtship indices that was observed in the magnetic field, indicating that CRY-signaling was necessary for the increase in male courtship activity. Genetically re-expressing wild-type *cry* in *cry-GAL4*-positive neurons restored the increase in the courtship index under the magnetic field conditions. Finally, artificial activation of *cry-GAL4*-positive large ventral lateral neurons (l-LNvs) and small ventral lateral neurons (s-LNvs) using *dTrpA1* also increased male courtship activity, serving to mimic the behavior of wild-type animals in a magnetic field environment. Together, our data suggest that *Drosophila melanogaster* may sense the magnetic field via a blue light-dependent CRY pathway in *cry-GAL4*-positive neurons, and this magnetic sensation may cause an increase in male courtship activity.

## Results

### Magnetic field increases male courtship activity

**Fig 1:**
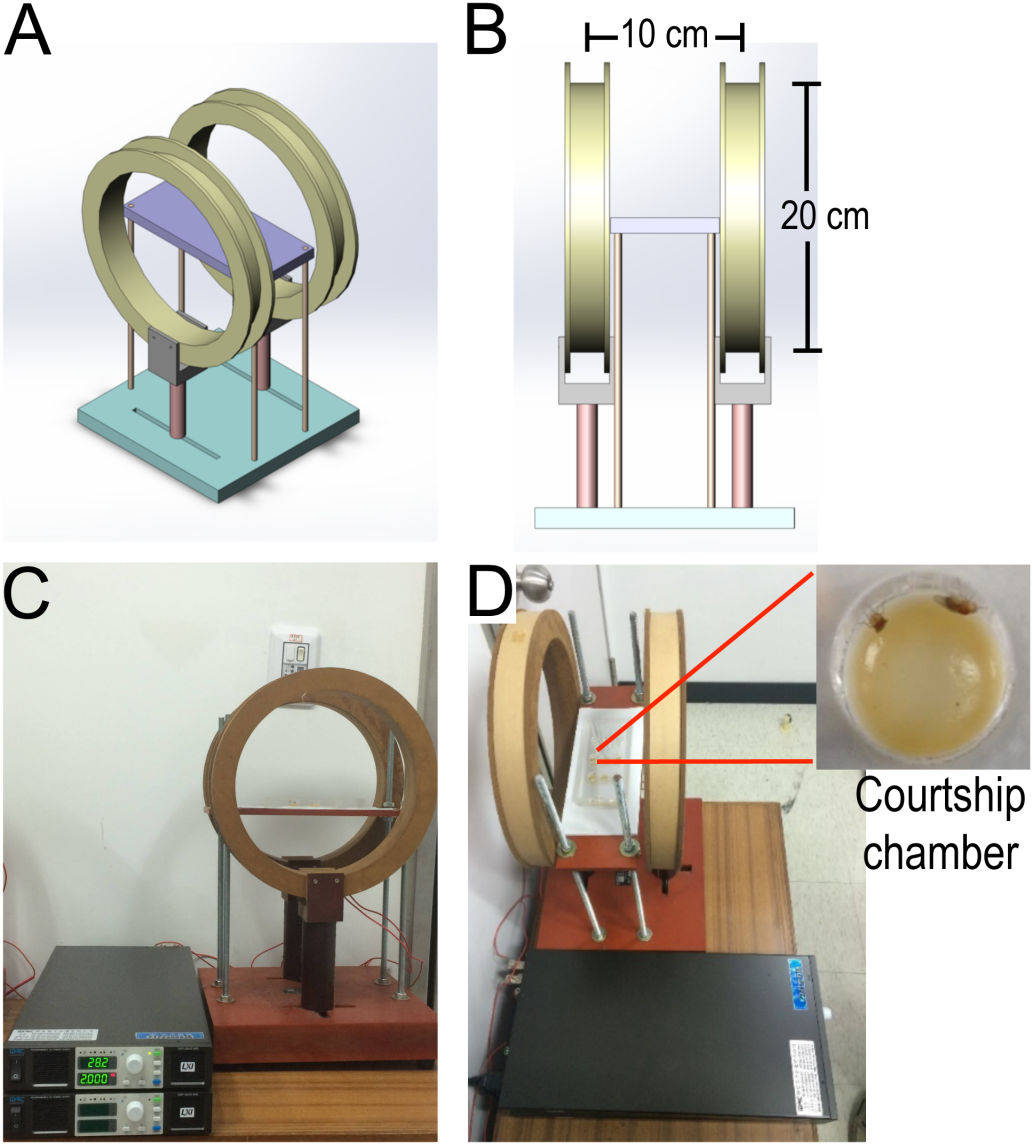
Fly courtship behavioral assay on a Helmholtz coil platform. (A-B) Diagrams of the Helmholtz coil platform. (C) Photograph of the Helmholtz coil platform, which is composed of two Helmholtz coil rings and a DC power supply. (D) The courtship chambers were placed in the middle of the Helmholtz coil platform, and a video camera on the top of the platform was used to record the fly courtship behaviors.

We developed an electromagnetic field stimulation platform using Helmholtz coils to generate a uniform magnetic field environment. Placement of the courtship behavior chamber on the electromagnetic platform allowed us to observe courtship while manipulating magnetic field conditions (Fig 1). The strength of the magnetic field was controlled by the input currents and determined by a Gaussmeter (see Methods section for details). Uniform magnetic fields (10, 20, 40, 60, and 80 G) were used.

**Fig 2:**
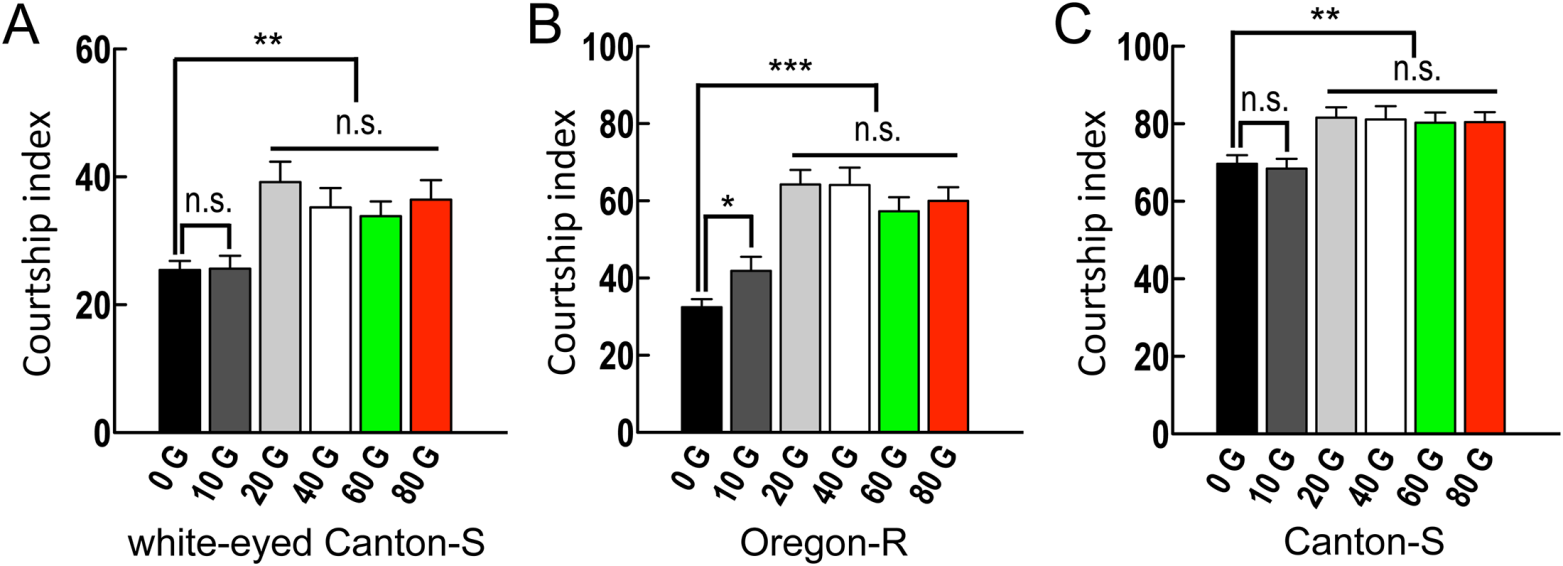
Courtship indices under magnetic fields of various strengths. Courtship activities displayed by different *Drosophila* strains in various magnetic field strengths (10, 20, 40, 60, and 80 G) in full-spectrum light. The bars show the courtship indices of the three wild-type flies under different magnetic field intensities. (A) white-eyed Canton-S, (B) red-eyed Oregon-R, and (C) red-eyed Canton-S. Each value represents the mean + SEM (n ≥ 8; *p < 0.05, **p < 0.01, and ***p < 0.001; n.s., not statistically significant; ANOVA followed by Tukey’s tests).

Interestingly, we found that the wild-type flies, including the white-eyed Canton-S, red-eyed Oregon-R, and red-eyed Canton-S fly strains, all displayed increased courtship activities in enhanced magnetic fields (≥ 20 G). These results suggest that the courtship index increased when flies sensed the magnetic field and that differences in eye color did not substantially alter behavioral responses under increasing magnetic field strengths (Fig 2). The variable baselines of male courtship activity under a 0-Gauss magnetic field among different wild-type fly strains may be caused by differences in the *white* gene (Fig 2) [14–16]. To avoid having the different baselines of male courtship activity influence our conclusions, we compared the courtship indexes from the same fly in different magnetic fields or temperatures, except in the comparison of white-eyed Canton-S with *cry*^*b*^ or *cry*^*m*^ mutants (S1A Fig).

### Increase in male courtship activity in a magnetic field requires CRY signaling

**Fig 3:**
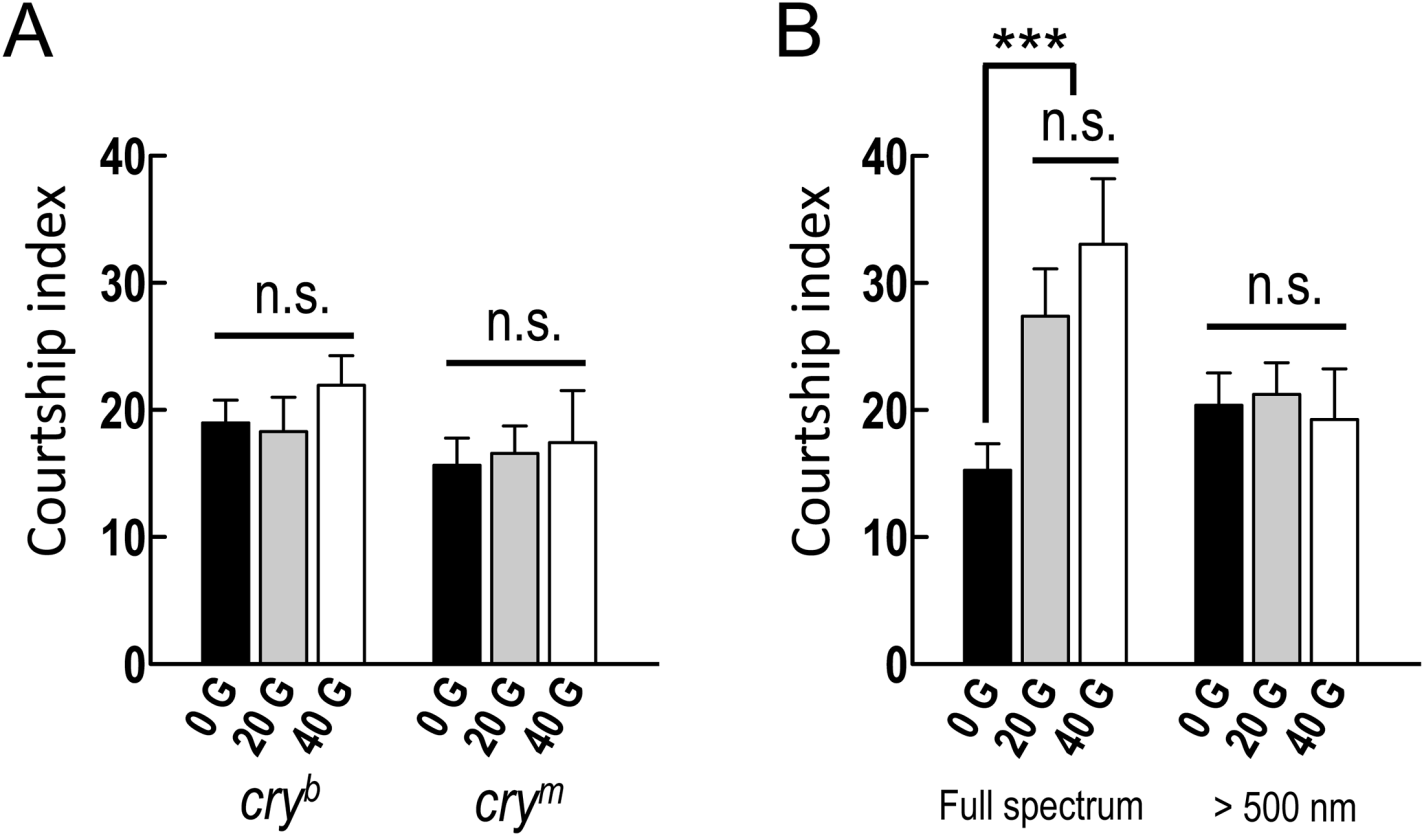
The increase in male courtship activity in a magnetic field is cryptochrome- (CRY-) and blue light-dependent. (A) Two CRY mutant flies did not show increased courtship indices in magnetic fields (20 G and 40 G) compared with the control (0 G). Each value represents the mean + SEM (n ≥ 9; n.s., not significant; ANOVA). Genotypes: (1) *w/Y; +/+; cry*^*b*^*/cry*^*b*^, (2) *w/Y; +/+; cry*^*m*^*/cry*^*m*^. (B) In white-eyed Canton-S male flies, the increase in courtship activity induced by the magnetic field was blue light-dependent. The bars show the courtship index values for naïve responses under full-spectrum light (left panel) and for light with wavelengths > 500 nm (right panel). Each value represents the mean + SEM (n ≥ 9; ***p < 0.001; n.s., not statistically significant; ANOVA followed by Tukey’s tests).

In order to examine whether the increase in courtship behavior could be attributed to altered CRY activity in magnetic field conditions, we performed additional courtship assays in *cry* mutants under enhanced magnetic field conditions. The *cry*^*b*^ mutation affects a highly conserved protein domain that is likely involved in FAD binding, which is necessary for both CRY and photolyase functions [17]. The *cry*^*m*^ mutation truncates the C-terminal domain of CRY, leaving the photolyase domain intact [18]. Under magnetic field conditions (20 or 40 G), the courtship activity in *cry*^*b*^ and *cry*^*m*^ mutants did not increase over that in the natural environment (0 G), suggesting that this behavioral phenotype results from magnetoreception through a CRY-dependent pathway (Fig 3A and S1A Fig). Because CRY-dependent magnetic responses are light-dependent, we examined wild-type flies under a long-pass filter that transmit light with wavelengths > 500 nm. We found that under this restricted-spectrum light, flies did not show a significant increase in courtship activity, even under the enhanced magnetic field conditions (Fig 3B and S1B Fig).

### CRY expression in *cry-GAL4*-positive neurons mediates the increase in male courtship activity in a magnetic field

To determine whether CRY expression in *cry-GAL4-*positive neurons was involved in increasing male courtship activity in an enhanced magnetic field, we used the GAL4/UAS system to evaluate the efficiency of knockdown in *UAS-cry*^*RNAi*^ flies (in which expression is under the control of the pan-neuronal driver *elav-GAL4*). The effectiveness of each *UAS-cry*^*RNAi*^ was validated using quantitative PCR (S2A Fig). Both manipulated flies (*elav-GAL4/UAS-cry*^*RNAi*^) showed significant knockdown in *cry* mRNA compared with the control group (*elav-GAL4/+*). We further tested whether targeted knockdown of *cry* expression in *cry-GAL4*-positive neurons would diminish increased courtship activity in a magnetic field. In the 20-G magnetic field, knockdown of CRYs using the *cry-GAL4* driver in *UAS-cry*^*RNAi*^ flies diminished the increases in male courtship activity caused by the magnetic field (S2B Fig).

**Fig 4:**
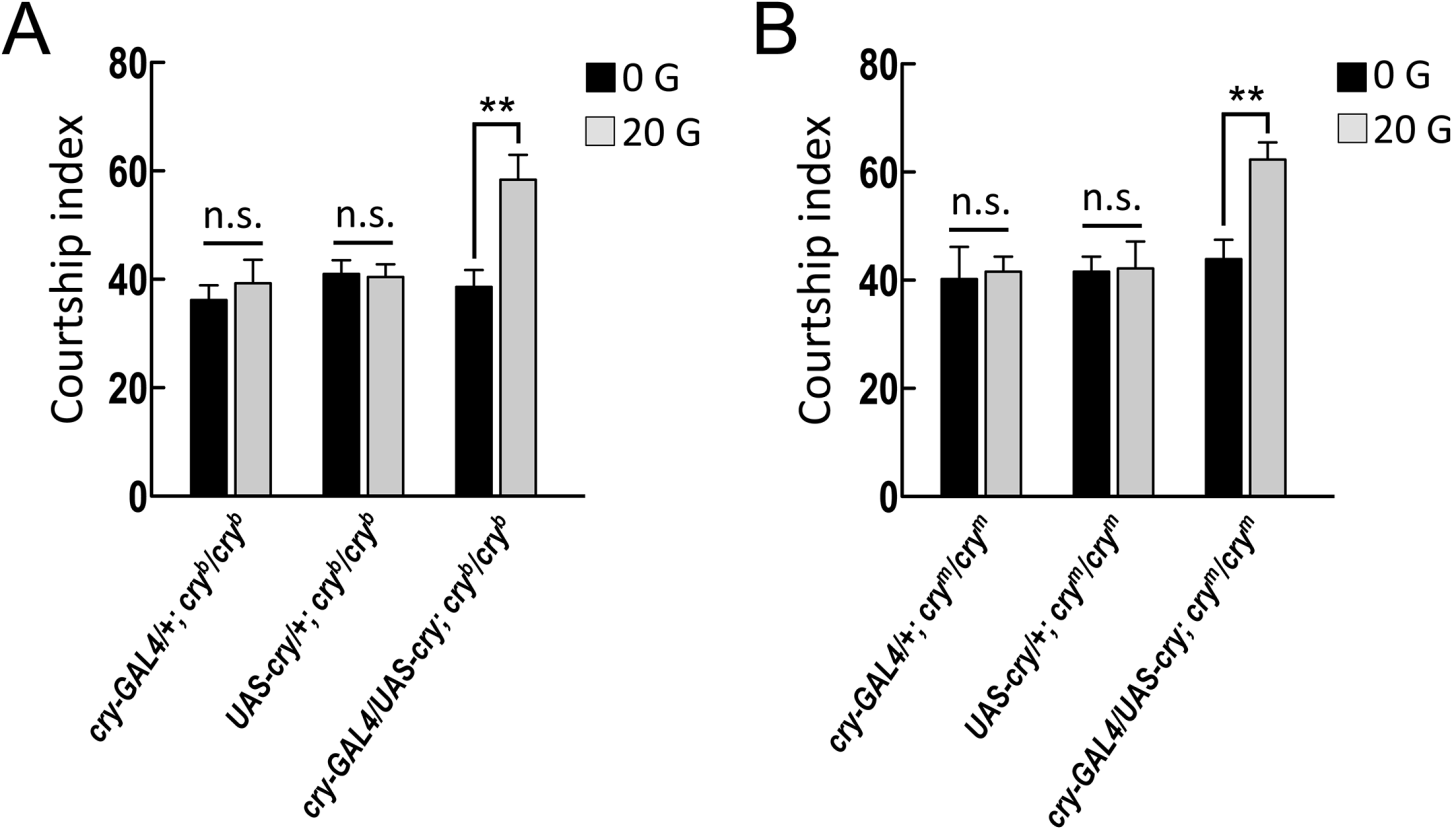
Expression of cryptochrome (CRY) with *cry-GAL4* restores the increase in courtship activity in *cry* mutants in a magnetic field. (A) In the 20-G magnetic field environment, overexpression of the *cry* transgene in *cry-GAL4-*positive neurons restored the increase in courtship activity (indicated by courtship index) that was observed in flies with the *cry*^*b*^ background, compared with the 0-G control. Each value represents the mean + SEM (n ≥ 9; **p < 0.01; n.s., not statistically significant; *t*-tests). Genotypes: (1) *w/Y; cry-GAL4/+; cry*^*b*^*/cry*^*b*^, (2) *w/Y; +/UAS-cry; cry*^*b*^*/cry*^*b*^, (3) *w/Y; cry-GAL4/UAS-cry; cry*^*b*^*/cry*^*b*^. (B) In the 20-G magnetic field, overexpression of the *cry* transgene in *cry-GAL4-*positive neurons restored the increase in courtship index that was observed in flies with the *cry*^*m*^ background compared with the 0-G control. Each value represents the mean + SEM (n ≥ 12; **p < 0.01; n.s., not statistically significant; *t*-tests). Genotypes: (1) *w/Y; cry-GAL4/+; cry*^*m*^*/cry*^*m*^, (2) *w/Y; +/UAS-cry; cry*^*m*^*/cry*^*m*^, (3) *w/Y; cry-GAL4/UAS-cry; cry*^*m*^*/cry*^*m*^.

Furthermore, we genetically re-expressed the wild-type *cry* transgene in *cry-GAL4*-positive neurons in *cry*^*b*^ or *cry*^*m*^ mutant backgrounds and evaluated the courtship behaviors of these flies in a 20-G magnetic field. *UAS-cry* transgene expression under the control of *cry-GAL4* in the *cry* mutants restored the increase in courtship activity in a 20-G magnetic field, suggesting that the expression of the CRYs in *cry-GAL4*-positive neurons is sufficient to increase courtship activity in a magnetic field (Fig 4).

### Activating l-LNvs and s-LNvs increases male courtship activity

**Fig 5:**
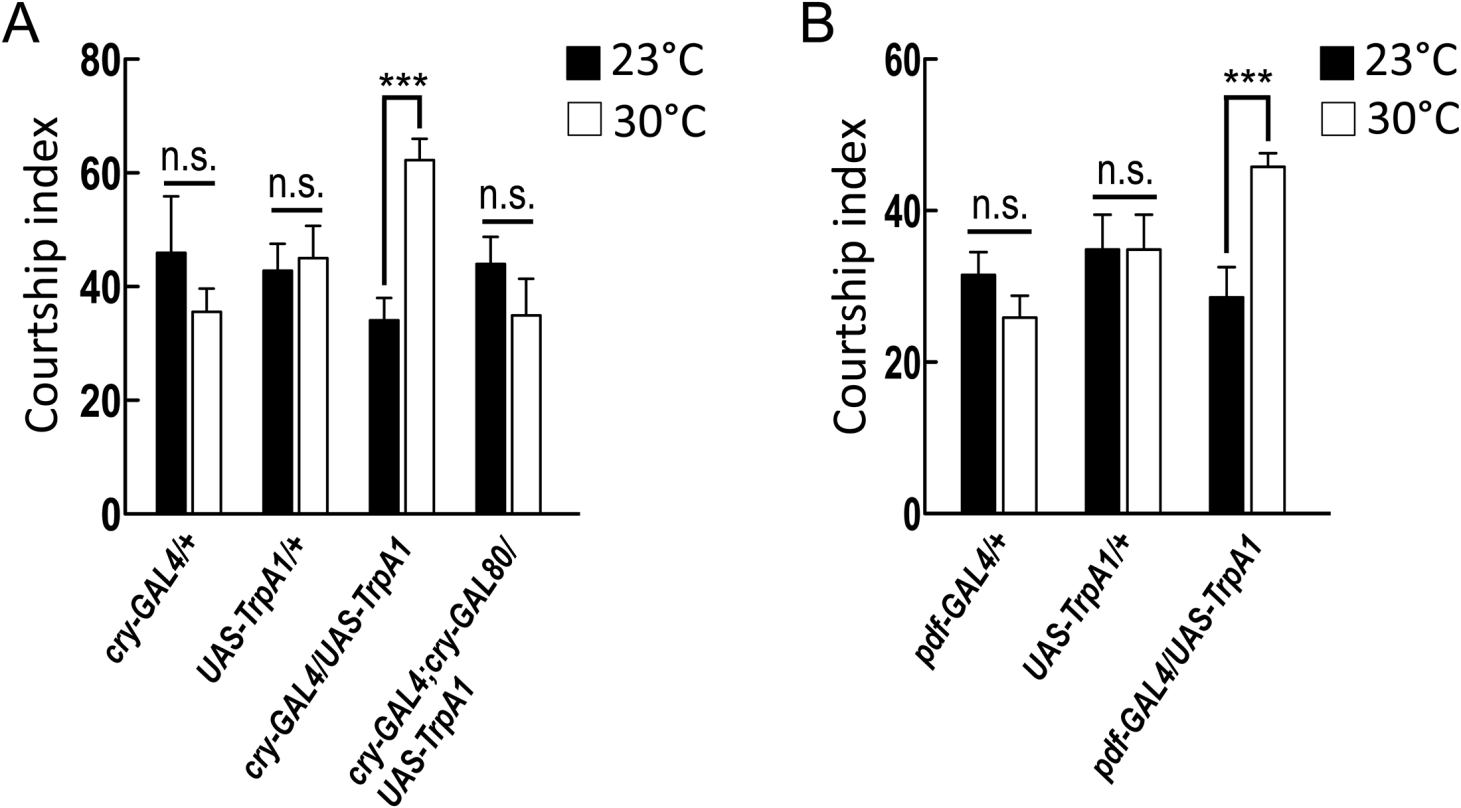
Activating large ventral lateral neurons (l-LNvs) and small ventral lateral neurons (s-LNvs) increases male courtship activity. (A) Activation of *cry-GAL4-*expressing neurons increased the male courtship index, whereas *cry-GAL80* suppressed this behavioral phenotype. Each value represents the mean + SEM (n ≥13; ***p < 0.001; n.s., not statistically significant; *t*-tests). Genotypes: (1) *w/Y; cry-GAL4/+; +/+*, (2) *w/Y; +/+; +/UAS-TrpA1*, (3) *w/Y; cry-GAL4/+; +/UAS-TrpA1*, (4) *w/Y; cry-GAL4/+; cry-GAL80/UAS-TrpA1*. (B) Activation of *pdf-GAL4-*expressing neurons increased the male courtship index. Each value represents the mean + SEM (n ≥ 8; ***p < 0.001; n.s., not statistically significant; *t*-tests). Genotypes: (1) *w/Y; pdf-GAL4/+; +/+*, (2) *w/Y; +/+; +/UAS-TrpA1*, (3) *w/Y; pdf-GAL4/+; +/UAS-TrpA1*.

Expression driven by *cry-GAL4* targets a small subsets of neurons in the fly brain, including the dorsal lateral neurons (LNds), l-LNvs, and s-LNvs, as well as neurons in small subsets of the ellipsoid body (S3A Fig and [11,12]). To examine whether the activation of CRY-positive neurons could increase courtship activity in male flies, we used the *cry-GAL4* driver to target the expression of the thermosensitive cation channel *dTrpA1* to CRY neurons and enabled their activation by increasing the temperature to 30°C. The manipulated male flies showed robust courtship activity that was higher in the 30°C group than in the 23°C group (Fig 5A).

Importantly, the increase in courtship activity was totally blocked when the *cry-GAL4* driver was combined with *cry-GAL80* to inhibit GAL4 expression in CRY neurons (Fig 5A and S3B Fig). *cry-GAL4* is expressed not only in a subset of clock neurons (LNds, l-LNvs, and s-LNvs) but also in the ellipsoid body of the deep brain (S3A Fig and [11,12]). Activating the ellipsoid body neurons via *TrpA1* under the control of the *VT4244-GAL4* driver did not increase courtship activity in male flies (S4 Fig).

We then used *pdf-GAL4*, expressed in l-LNvs and s-LNvs, to further evaluate the role of subsets of clock neurons in male flies’ courtship activity (S3C Fig) [19]. Genetically activating l-LNvs and s-LNvs via *TrpA1*, under the control of the *pdf-GAL4* driver, significantly increased courtship activity in male flies in the 30°C group over that of flies in the 23°C group. This finding suggests that the activation of l-LNvs and s-LNvs is sufficient to increase courtship activity in male flies (Fig 5B).

## Discussion

The courtship behavior of *Drosophila melanogaster* consists of several stereotypical behaviors that are performed by males in response to various target-derived sensory inputs. The courtship index is a quantitative expression of the duration of male flies’ courtship behavior and is measured as the percentage of time spent on courtship behavior throughout the experimental period. In the present study, we noted a quantitative increase in the courtship index when male flies were exposed to magnetic fields over 20 G (Fig 2). Enhanced magnetic field environments increased the male courtship index, an effect that requires functional CRY expression in *cry-GAL4* positive neurons. In *Drosophila*, CRY is the primary circadian photoreceptor and is expressed in a subset of clock neurons, the LNds, l-LNvs, and s-LNvs, as well as in subsets of the ellipsoid body neurons (S3A Fig and [11,12]).

Previous studies have demonstrated that magnetosensitivity in *Drosophila* requires blue light-dependent CRY expression and is mediated through the radical-pair mechanism, with light-activated flavin-based photoreceptors acting as sensors for electromagnetic fields [8,13]. In the present study, genetically activating the l-LNvs and s-LNvs but not the ellipsoid body neurons via *UAS-TrpA1* increased the male courtship index (Fig 5 and S3 Fig). This finding suggests that blue light-induced CRY-dependent magnetosensation could trigger the increase in male courtship activity by altering the activity in the l-LNvs and s-LNvs.

The gene *cry*^*b*^ contains a missense mutation that affects a highly conserved FAD-binding domain, which is necessary for CRY and photolyase function [17]. The *cry*^*m*^ mutation changes the Arg524 codon into a stop codon, which truncates the C-terminal domain of CRY [18]. The behavioral results we recorded for *cry*^*b*^ and *cry*^*m*^ mutant flies suggest that the increase in male courtship activity after transient exposure to the magnetic field requires not only the FAD-binding domain but also the C-terminal region of the CRY protein (Fig 3A and S1A Fig).

A recent study demonstrated that deletion of the CRY C-terminal region disrupted electromagnetic field–induced negative geotaxis impairments [20]. In the current study, we propose that both the FAD-binding domain and the C-terminal region of CRY are required for the increase in male courtship activity in magnetic fields. The CRY-mediated light response, which increases the frequency of spontaneous firing, caused a cell autonomous, Flavin redox–based mechanism that depends on potassium channel conductance [21,22]. Here, we did not observe a significant increase in courtship activity under full-spectrum light in the absence of a magnetic field (Fig 3B and S1B Fig). The magnetic field may affect male courtship activity by altering the neural activity via a CRY-dependent pathway. Whether the magnetic field alters the firing of the action potential of CRY-expressing neurons in the blue-light environment remains uncertain.

Interestingly, genetically activating the l-LNvs and s-LNvs with *TrpA1* increased the male courtship index in the absence of a magnetic field (Fig 5), suggesting that the magnetic field may affect male courtship activity by altering the activity of *cry-GAL4*-positive l-LNvs and s-LNvs. More studies are needed to elucidate the physiological mechanisms by which the CRY signaling mediates courtship behavior in male flies.

## Materials and Methods

### Fly strains

Flies were raised on standard cornmeal media at 25°C and 60% relative humidity under a 12-h light:12-h dark cycle. The white-eyed Canton-S [23], *elav-GAL4*, *cry-GAL4*, *UAS-mCD8*::*GFP; UAS-mCD8*::*GFP*, *pdf-GAL4*, *UAS-TrpA1*, *pdf-GAL80*, and *VT4244-GAL4* lines were obtained from Dr. Ann-Shyn Chiang. The Oregon-R and red-eyed Canton-S strains were obtained from Dr. Li-Mei Pai. The *cry*^*b*^, *cry*^*m*^, and *UAS-cry* lines were gifts from Dr. Patrick Emery. The *UAS-cry*^*RNAi*^ (*v7238* and *v7239*) flies were obtained from the Vienna *Drosophila* Resource Center (VDRC).

### Helmholtz coil platform

Two Helmholtz coils were used to build the platform. Each coil was wound with 332 copper wires and was 20 cm in diameter. The distance between the two coil rings was 10 cm (Fig 1B). The platform generated a uniform magnetic field of different intensities (10 G, 20 G, 40 G, 60 G, and 80 G) by using a DC power supply to input different currents and voltages. The intensity of the magnetic field was measured using a Gaussmeter (Sypris Solutions, Inc., Model #7030, California, USA), with a hall probe manipulator, to evaluate the intensity of the magnetic field in the courtship chamber (Fig 1D).

### Courtship assay

To evaluate courtship behavior, one male and one virgin female fly were placed in a chamber on the magnetic apparatus, and their courtship behaviors were recorded with a video camera. The courtship behavioral assay followed procedures established by a previous study [24]. Naïve males with no pre-test social experience were collected on the day of eclosion and kept individually in test tubes in a 25°C incubator with an L/D cycle. Target females were stored in groups (20 females per vial). The courtship assays were conducted between 2 and 6 h into the light cycle in the courtship chamber (1.2 cm diameter × 0.8 cm high), which contained a layer of yeast media. The flies were anesthetized with mild CO_2,_ and both test males and target females (female Canton-S, 3 days after eclosion) were transferred to the behavior chamber, where the magnetic field was established. The courtship index is defined as the percentage of time that the tested male spent courting the target female during a 10-min recording period (e.g., tapping, following, vibrating wings, and attempting to copulate). For the *TrpA1* studies, all of the flies were raised at 23°C before the experiments, and were placed in a 23°C or 30°C environment 10 min before and during the courtship behavioral assays.

### Whole-mount immunostaining

*Drosophila* brains were dissected in phosphate-buffered saline (PBS) and fixed in 4% paraformaldehyde for 20 min at room temperature. After fixation, the brain samples were incubated in PBS containing 1% Triton X-100 and 10% normal goat serum (PBS-T) and degassed in a vacuum chamber to expel tracheal air with six cycles of depressurizing to 270 mm Hg followed by holding for 10 min). Next, the brain samples were blocked and penetrated in PBS-T at 25°C for 2 h and then incubated in PBS-T containing mouse 4F3 anti-discs large (DLG) monoclonal antibody (diluted 1:10, Developmental Studies Hybridoma Bank, University of Iowa) at 25°C for one day. After the samples were washed in PBS-T three times, the samples were incubated in a biotinylated goat anti-mouse antibody (diluted 1:200, Molecular Probes, Thermo Fisher Scientific) at 25°C for one day. Next, the brain samples were washed and incubated in Alexa Fluor 635-conjugated streptavidin (diluted 1:500, Molecular Probes) at 25°C for one day. After extensive washing, the brain samples were cleared and mounted in FocusClear (CelExplorer) for confocal imaging.

### Confocal microscopy

Fly brain samples were imaged using a Zeiss LSM 700 confocal microscope with a 40X C-Apochromat water-immersion objective lens. To overcome the limited field of view, the brain samples were imaged twice, one for each hemisphere, with an overlap in between. We then combined the two parallel image stacks into a single dataset with ZEN image-processing software, using the overlapping region to align the two stacks.

### Statistics

All data were analyzed parametrically with Prism 5 statistical software (GraphPad). Data were evaluated by one-way analysis of variance (ANOVA) followed by Tukey’s multiple comparisons tests or evaluated by paired *t*-tests. All data are presented as the mean + standard error of the mean (SEM).

## Supporting Information

S1 Fig Behavioral control experiments in Fig 3. (A) Compared with *cry*^*b*^ or *cry*^*m*^ mutants, white-eyed Canton-S did not show a significant difference in courtship activity in a 0-Gauss environment (left panel), but these flies significantly increased their courtship activity in a 20-Gauss magnetic field (right panel). Each value represents the mean + SEM (n ≥ 9; ***p < 0.001, n.s., not significant; ANOVA followed by Tukey’s tests). Genotypes: (1) *w/Y; +/+; +/+*, (2) *w/Y; +/+; cry*^*b*^*/cry*^*b*^, (3) *w/Y; +/+; cry*^*m*^*/cry*^*m*^. (B) A restricted wavelength of light (> 500 nm) did not affect normal courtship activity in white-eyed Canton-S male flies (n ≥ 10; n.s., not significant; *t*-tests).
(TIF)

S2 Fig Knockdown of CRY with *cry-GAL4* diminishes the increase in courtship activity caused by a magnetic field. (A) Effectiveness of knockdown in the *UAS-cry*^*RNAi*^ line used in this study. Quantitative polymerase chain reaction (PCR) analysis showed that there was less targeted mRNA in the *elav-GAL4/UAS-cry*^*RNAi*^ *(v7238)* and *elav-GAL4/UAS-cry*^*RNAi*^ *(v7239)* flies than in the control *elav-GAL4/+* flies. The results were normalized to the relative amount of 60S ribosomal protein L32 (RpL32). Each value represents the mean + SEM. (n ≥ 3). The forward and reverse primers used were 5′-AGGGTATAGCCCTAATTCCCG-3′ and 5′-GCATCCGATTGTAACCCACATT-3′, respectively. Genotypes: (1) *w/elav-GAL4; +/+; +/+*, (2) *w/elav-GAL4; +/+; +/UAS-cry*^*RNAi*^*(v7238)*, (3) *w/elav-GAL4; +/+; +/UAS-cry*^*RNAi*^*(v7239)*. (B) RNAi-mediated knockdown of *cry* in *cry-GAL4-*expressing neurons inhibited the increase in courtship indices in the 20-G magnetic field, compared with the 0-G control. Each value represents the mean + SEM (n ≥ 14; *p < 0.05, **p < 0.01, and ***p < 0.001; n.s., not statistically significant; *t*-tests). Genotypes: (1) *w/Y; cry-GAL4/+; +/+*, (2) *w/Y; +/+; +/UAS-cry*^*RNAi*^*(v7238)*, (3) *w/Y; +/+; +/UAS-cry*^*RNAi*^*(v7239)*, (4) *w/Y; cry-GAL4/+; +/UAS-cry*^*RNAi*^*(v7238)*, (5) *w/Y; cry-GAL4/+; +/UAS-cry*^*RNAi*^*(v7239)*.
(TIF)

S3 Fig Green fluorescent protein (GFP) expression patterns driven by the GAL4 lines used in the present study. (A) GFP expression pattern (green) in the brain of a *cry-GAL4 > UAS-mCD8*::*GFP; UAS-mCD8*::*GFP* male individual. Genotype: *w/Y; cry-GAL4/UAS-mCD8*::*GFP; +/UAS-mCD8*::*GFP*. (B) GFP expression pattern (green) in the brain of a *cry-GAL4; cry-GAL80 > UAS-mCD8*::*GFP; UAS-mCD8*::*GFP* male individual. Genotype: *w/Y; cry-GAL4/UAS-mCD8*::*GFP; cry-GAL80/UAS-mCD8*::*GFP*. (C) GFP expression pattern (green) in the brain of a *pdf-GAL4 > UAS-mCD8*::*GFP; UAS-mCD8*::*GFP* male individual. Genotype: *w/Y; pdf-GAL4/UAS-mCD8*::*GFP; +/UAS-mCD8*::*GFP*. The brains were immunostained with anti-DLG antibody (magenta). The scale bars represent 50 μm.
(TIF)

S4 Fig Activating ellipsoid body neurons did not increase male courtship activity. (A) Green fluorescent protein (GFP) expression pattern (green) in the brain of a *VT4244-GAL4 > UAS-mCD8*::*GFP; UAS-mCD8*::*GFP* male individual. Genotype: *w/Y; +/UAS-mCD8*::*GFP; VT4244-GAL4/UAS-mCD8*::*GFP*. The brain was immunostained with anti-DLG antibody (magenta). The scale bar represents 50 μm. (B) Activating *VT4244-GAL4*-expressing neurons did not increase the courtship index. Each value represents the mean + SEM (n ≥ 6; n.s., not statistically significant; *t*-tests). Genotypes: *w/Y; +/+; VT4244-GAL4/UAS-TrpA1*.
(TIF)
