# Supplementary figures and images for: Magnetoreception Regulates Male Courtship Activity in Drosophila
Source: PLoS One. 2016 May 19;11(5):e0155942. doi: 10.1371/journal.pone.0155942 (PMC4873040; doi:10.1371/journal.pone.0155942)

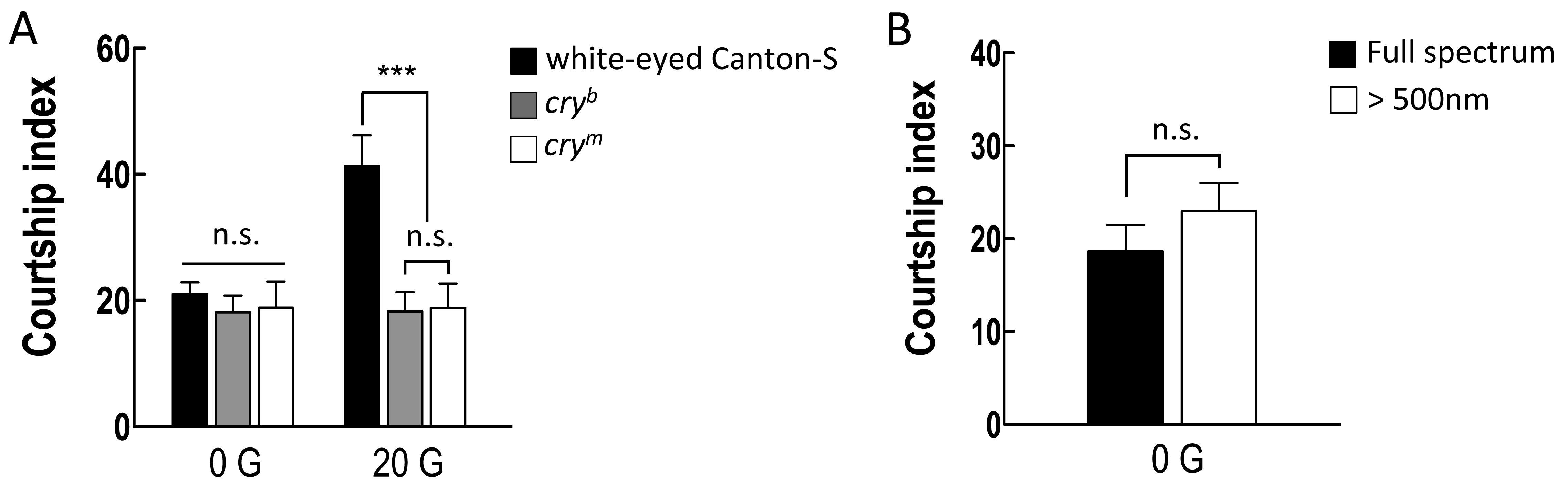

Supplement: S1 Fig — (A) Compared with cryb or crym mutants, white-eyed Canton-S did not show a significant difference in courtship activity in a 0-Gauss environment (left panel), but these flies significantly increased their courtship activity in a 20-Gauss magnetic field (right panel). Each value represents the mean + SEM (n ≥ 9; ***p < 0.001, n.s., not significant; ANOVA followed by Tukey’s tests). Genotypes: (1) w/Y; +/+; +/+, (2) w/Y; +/+; cryb/cryb, (3) w/Y; +/+; crym/crym. (B) A restricted wavelength of light (> 500 nm) did not affect normal courtship activity in white-eyed Canton-S male flies (n ≥ 10; n.s., not significant; t-tests). (TIF) [file pone.0155942.s001.tif]

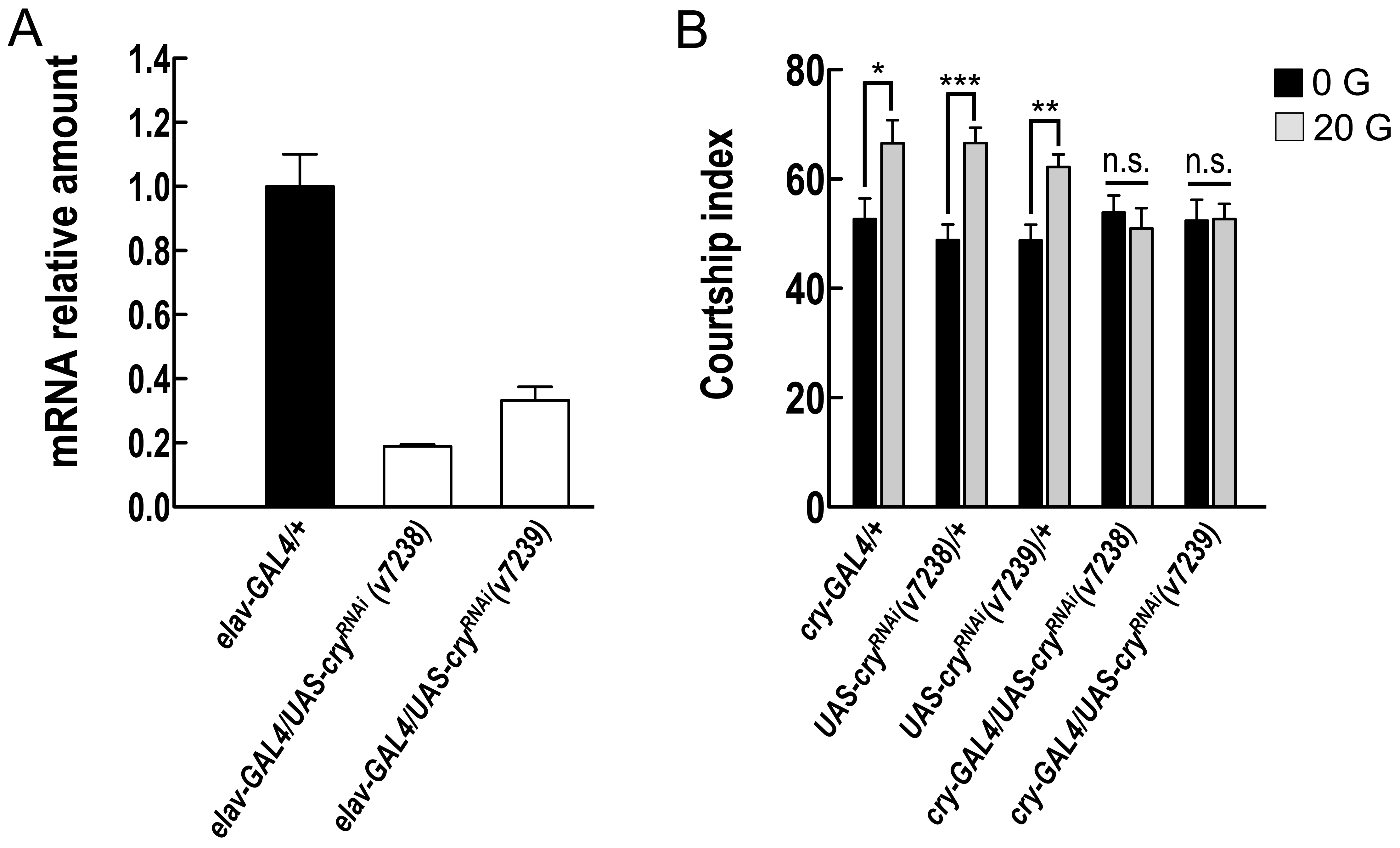

Supplement: S2 Fig — (A) Effectiveness of knockdown in the UAS-cryRNAi line used in this study. Quantitative polymerase chain reaction (PCR) analysis showed that there was less targeted mRNA in the elav-GAL4/UAS-cryRNAi (v7238) and elav-GAL4/UAS-cryRNAi (v7239) flies than in the control elav-GAL4/+ flies. The results were normalized to the relative amount of 60S ribosomal protein L32 (RpL32). Each value represents the mean + SEM. (n ≥ 3). The forward and reverse primers used were 5′-AGGGTATAGCCCTAATTCCCG-3′ and 5′-GCATCCGATTGTAACCCACATT-3′, respectively. Genotypes: (1) w/elav-GAL4; +/+; +/+, (2) w/elav-GAL4; +/+; +/UAS-cryRNAi(v7238), (3) w/elav-GAL4; +/+; +/UAS-cryRNAi(v7239). (B) RNAi-mediated knockdown of cry in cry-GAL4-expressing neurons inhibited the increase in courtship indices in the 20-G magnetic field, compared with the 0-G control. Each value represents the mean + SEM (n ≥ 14; *p < 0.05, **p < 0.01, and ***p < 0.001; n.s., not statistically significant; t-tests). Genotypes: (1) w/Y; cry-GAL4/+; +/+, (2) w/Y; +/+; +/UAS-cryRNAi(v7238), (3) w/Y; +/+; +/UAS-cryRNAi(v7239), (4) w/Y; cry-GAL4/+; +/UAS-cryRNAi(v7238), (5) w/Y; cry-GAL4/+; +/UAS-cryRNAi(v7239). (TIF) [file pone.0155942.s002.tif]

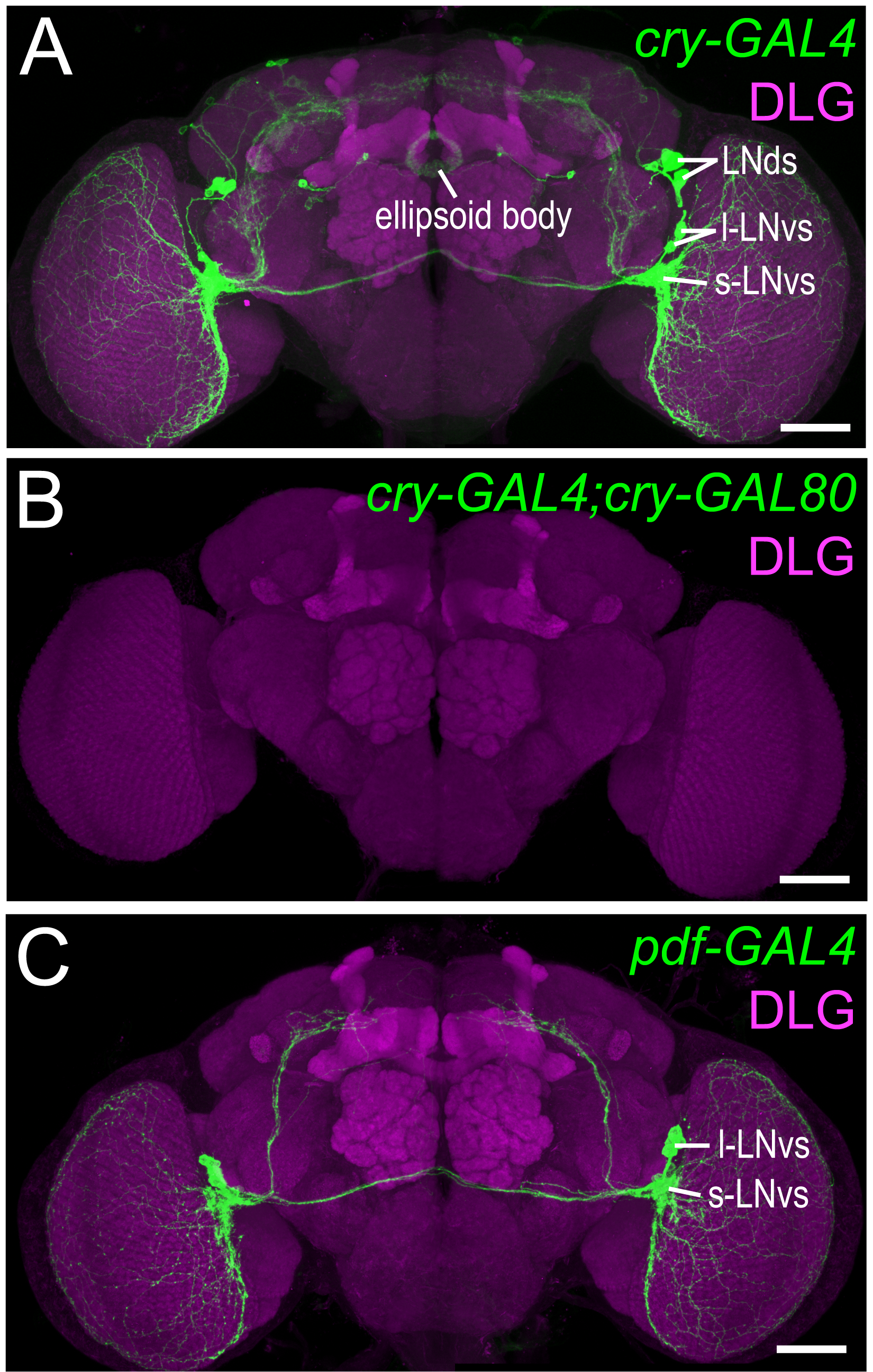

Supplement: S3 Fig — (A) GFP expression pattern (green) in the brain of a cry-GAL4 > UAS-mCD8::GFP; UAS-mCD8::GFP male individual. Genotype: w/Y; cry-GAL4/UAS-mCD8::GFP; +/UAS-mCD8::GFP. (B) GFP expression pattern (green) in the brain of a cry-GAL4; cry-GAL80 > UAS-mCD8::GFP; UAS-mCD8::GFP male individual. Genotype: w/Y; cry-GAL4/UAS-mCD8::GFP; cry-GAL80/UAS-mCD8::GFP. (C) GFP expression pattern (green) in the brain of a pdf-GAL4 > UAS-mCD8::GFP; UAS-mCD8::GFP male individual. Genotype: w/Y; pdf-GAL4/UAS-mCD8::GFP; +/UAS-mCD8::GFP. The brains were immunostained with anti-DLG antibody (magenta). The scale bars represent 50 μm. (TIF) [file pone.0155942.s003.tif]

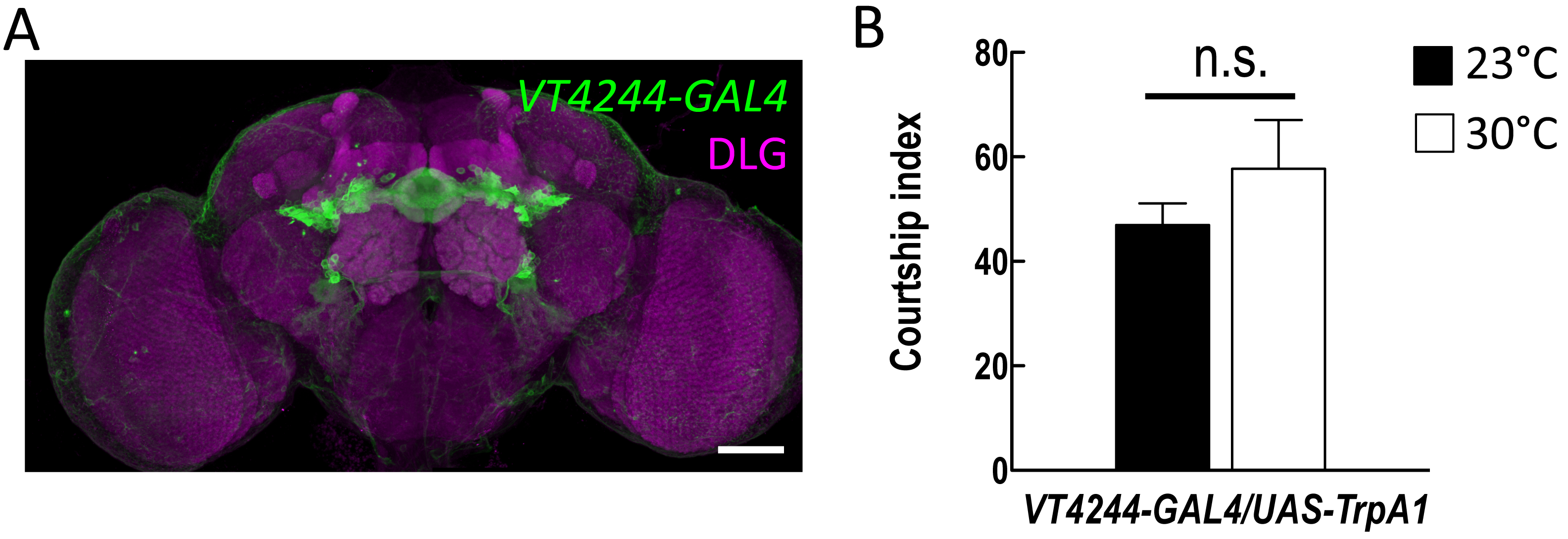

Supplement: S4 Fig — (A) Green fluorescent protein (GFP) expression pattern (green) in the brain of a VT4244-GAL4 > UAS-mCD8::GFP; UAS-mCD8::GFP male individual. Genotype: w/Y; +/UAS-mCD8::GFP; VT4244-GAL4/UAS-mCD8::GFP. The brain was immunostained with anti-DLG antibody (magenta). The scale bar represents 50 μm. (B) Activating VT4244-GAL4-expressing neurons did not increase the courtship index. Each value represents the mean + SEM (n ≥ 6; n.s., not statistically significant; t-tests). Genotypes: w/Y; +/+; VT4244-GAL4/UAS-TrpA1. (TIF) [file pone.0155942.s004.tif]
